# Impact of Chlorine Dioxide Gas Sterilization on Nosocomial Organism Viability in a Hospital Room

**DOI:** 10.3390/ijerph10062596

**Published:** 2013-06-21

**Authors:** John J. Lowe, Shawn G. Gibbs, Peter C. Iwen, Philip W. Smith, Angela L. Hewlett

**Affiliations:** 1 Department of Environmental, Agricultural & Occupational Health, University of Nebraska Medical Center College of Public Health, Omaha, NE 68198, USA; E-Mail: sgibbs@unmc.edu; 2 Department of Pathology and Microbiology, University of Nebraska Medical Center College of Medicine, Omaha, NE 68198, USA; E-Mail: piwen@unmc.edu; 3 Section of Infectious Diseases, University of Nebraska Medical Center College of Medicine, Omaha, NE 68198, USA; E-Mails: pwsmith@unmc.edu (P.W.S.); alhewlett@unmc.edu (A.L.H.)

**Keywords:** chlorine dioxide, sterilization, gas, nosocomial, hospital

## Abstract

To evaluate the ability of ClO_2_ to decontaminate pathogens known to cause healthcare-associated infections in a hospital room strains of *Acinetobacter baumannii*, *Escherichia coli*, *Enterococcus faecalis*, *Mycobacterium smegmatis*, and *Staphylococcus aureus* were spot placed in duplicate pairs at 10 sites throughout a hospital room and then exposed to ClO_2_ gas. Organisms were collected and evaluated for reduction in colony forming units following gas exposure. Six sterilization cycles with varied gas concentrations, exposure limits, and relative humidity levels were conducted. Reductions in viable organisms achieved ranged from 7 to 10-log reductions. Two sterilization cycles failed to produce complete inactivation of organisms placed in a bathroom with the door closed. Reductions of organisms in the bathroom ranged from 6-log to 10-log reductions. Gas leakage between hospital floors did not occur; however, some minor gas leakage from the door of hospital room was measured which was subsequently sealed to prevent further leakage. Novel technologies for disinfection of hospital rooms require validation and safety testing in clinical environments. Gaseous ClO_2_ is effective for sterilizing environmental contamination in a hospital room. Concentrations of ClO_2_ up to 385 ppm were safely maintained in a hospital room with enhanced environmental controls.

## 1. Introduction

The Centers for Disease Control and Prevention (CDC) estimates that 1.7 million hospitalized patients develop a hospital-associated infection (HAI) each year in the United States. These infections account for $4.5 to $6.5 billion in excess healthcare costs in the U.S. each year [1]. Studies have demonstrated environmental contamination may be a risk factor for acquisition of hospital-associated pathogens [2,3,4,5,6]. Outbreak and terminal cleaning investigations involving bacterial and viral organisms have focused on the hospital environment as a potential source of transmission and environmental cleaning efforts have been shown to play an important role in control of these outbreaks [7,8,9]. Although routinely employed, manual application of liquid sterilization chemicals is time consuming, labor intensive and prone to errors, thus prompting investigations into alternative methods for environmental disinfection [10,11,12]. Application of fumigating agents and ultraviolet (UV) light technologies have been explored as alternatives to manual disinfection for entire rooms. Fumigating agents such as ozone and hydrogen peroxide vapor have been evaluated in clinical environments as well as UV technologies [13,14,15]. These studies evaluated the efficacy of non-manual methods in addition to manual cleaning to reduce bacterial capture from hospital surfaces often during an active HAI outbreak.

This study evaluates the ability of chlorine dioxide to reduce a variety of nosocomial organisms throughout a hospital room. Chlorine dioxide (ClO_2_) is an EPA-registered gaseous sterilant that was utilized to decontaminate federal buildings during the 2001 anthrax attacks [16,17]. As a gas, ClO_2_ is ideal for penetrating the small spaces found in patient care rooms. However, the significant relative humidity requirement for gaseous ClO_2_ decontamination with respect to bacterial spore inactivation may limit the benefit of penetrability, gaseous ClO_2_ has not been tested in the healthcare environment. This study evaluated the ability of gaseous ClO_2_ to decontaminate pathogens known to cause healthcare-associated infections on surfaces in a hospital room.

## 2. Experimental Section

### 2.1. Setting

A 20 m^2^ hospital room in the Nebraska Biocontainment Patient Care Unit (NBPCU), located at the University of Nebraska Medical Center, was selected to test whole room disinfection using ClO_2_. The rooms in this unit were chosen for evaluation because of the NBPCU’s unique construction and safety features common to biosafety level 3 laboratories [18]. The NBPCU is at negative pressure with an average of 23 air exchanges per hour with controlled access making it ideal for testing potentially hazardous disinfection technologies.

### 2.2. Organisms

Eight organisms were selected to test ClO_2_ sterilization in the hospital room: highly drug-resistant wild type *Acinetobacter baumannii* BC 9782, *Escherichia coli* ATCC 25922, *Escherichia coli* ATCC 51446, *Enterococcus faecalis* ATCC 29212, vancomycin-resistant *Enterococcus faecalis* ATCC 51299*, Mycobacterium smegmatis ATCC 14468*, *Staphylococcus aureus* ATCC 25323 and methicillin-resistant *Staphylococcus aureus* ATCC 43300. *Acinetobacter baumannii* BC 9782 is resistant to multiple classes of antimicrobial agents including all aminoglycosides, cephalosporins, fluoroquinolones, broad-spectrum penicillins, trimethoprim-sulfamethoxazole, and tetracycline, but is susceptible to carbapenems. Concentrated organism stocks, 3 × 10^10^ colony forming units/mL, were established using standard culturing methods for each organism [15,19,20,21]. Organism stock concentrations were measured using Siemens Microscan Turbidity Meter (Siemens Healthcare Diagnostics, Glasgow, DE, USA). Organism stocks were maintained at 4 °C for a maximum of 14 days.

The *A. baumannii* and *S. aureus* strains were grown on TSA plates and incubated at 37 °C for 24 h. *S. aureus* was incubated with 5% CO_2_ and the *A. baumannii* was incubated at ambient air. Isolated colonies were then inoculated into 100 mL of TSB and incubated at 37 °C for 24 h. The broth culture was then washed by centrifugation twice using dH_2_O. Following the second centrifugation, the pellet was diluted in phosphate buffer saline PBS and adjusted to concentration. 

Both *E. coli* and *E. faecalis* were prepared on brain heart infusion (BHI, Remel Labs, Lenexa, KS, USA) agar plates and incubated at 37 °C for 24 h. Isolated colonies were used to inoculate 100 mL BHI broth and incubated at 37 °C for 24 h. Liquid cultures were twice centrifuged to pellet cells and washed with dH_2_O. Following the second centrifugation, the pellet was diluted in phosphate buffer saline PBS and adjusted to concentration.

The *M. smegmatis* strain was grown on TSA plates containing 5% sheep blood (S-TSA) and incubated at 37 °C with 5% CO_2_ for 24 h. Isolated colonies were inoculated into 100 mL TSB containing 5% sheep blood and incubated at 37 °C with 5% CO_2_ for 24 h. Broth cultures were twice centrifuged to pellet cells and washed with dH_2_O. Pellets were diluted in PBS and adjusted to concentration. 

**Figure 1:**
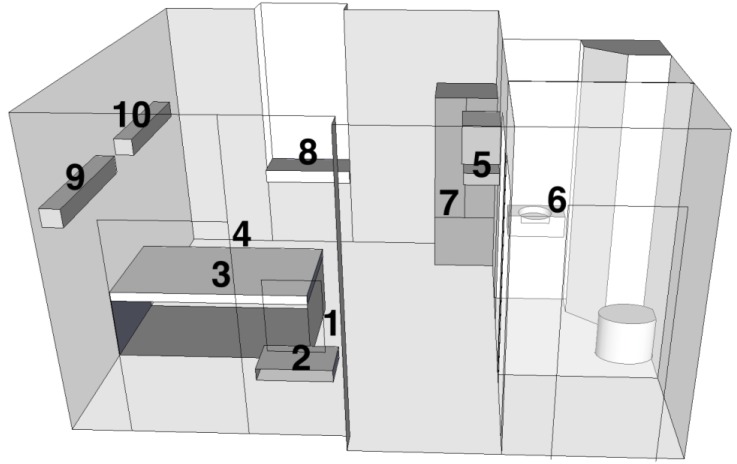
Organism spot placement sites utilized for hospital room chlorine dioxide environmental decontamination evaluation.

Commercially prepared *Bacillus atrophaeus* ATCC 9372 spore strip (Raven Labs, Omaha, NE, USA) biological indicators were also used to validate ClO_2_ decontaminations. *Bacillus atrophaeus* spore strip biological indicators are the standard for ClO_2_ decontamination validation. Each biological indicator strip was impregnated with a median value of 10^6^ spores and was contained in sterile Tyvek envelopes for aseptic processing. A 300 μL volume of each organism stock was pipetted into an empty well of a quad plate and transported to the hospital room in a hard sided container. Organism duplicate pairs were placed at 10 locations (Table 1, Figure 1) in the hospital room to assess complete gas penetration and one location in a control room. Control organisms were processed identically to exposure organisms. Log reductions were calculated from comparison of concentrations of control and exposure samples. Control and exposure concentrations were measured by serial dilution plate counts.

**Table 1:** Organism spot placement sites utilized for hospital room chlorine dioxide environmental decontamination evaluation.

Site #	Description	Meters from:		
		Injection Site	Floor	Ceiling
1	Floor next to injection tubing	0.3	0.0	3.3
2	Small wall mounted countertop	1.3	1.0	2.3
3	On top of bed mattress	1.6	1.3	2.0
4	Floor	2.7	0.0	3.3
5	Between wall mounted TV and VCR	3.0	2.3	1.0
6	Top of sink in bathroom	3.3	1.3	2.0
7	Inside metal cabinet	3.7	1.0	2.3
8	On window countertop	4.0	1.3	2.0
9	Top of wall mounted light fixture	4.3	2.7	0.7
10	Top of wall mounted light fixture	5.3	2.7	0.7

### 2.3. Chlorine Dioxide Sterilization

A total of six sterilization cycles were evaluated in the hospital room. Prior to each cycle the hospital room was sealed with duct tape and plastic sheeting from the outside to limit gas leakage into the hallway and adjacent rooms. The ClO_2_ generator, Minidox-M Decontamination System (Clordisys Solutions, Inc. Lebanon, NJ, USA), was placed in the hallway as a precaution to mitigate potential ClO_2_ leakage and two polyvinylidene fluoride tube lines were placed from the generator into the room through the space under the entry door. The tube lines allowed delivery of ClO_2_ gas into the room and air sampling of the room to monitor gas concentrations. Gas concentrations inside the room were continuously monitored by the generator. Two personnel were required at all times during the sterilization process to monitor the generator, adjacent areas and floors for leaks. Once the sterilization procedure was complete, the ClO_2_ gas was then evacuated from the hospital room using two activated charcoal scrubbers. One scrubber was operated inside the hospital room and one scrubber was located in the adjacent hallway.

### 2.4. Safety Considerations

Study personnel carried fit-tested full-face respirators equipped with acid gas filters. Adjacent areas and floors were continuously monitored for ClO_2_ gas with a hand held Portasens II detector (Analytical Technology, Inc. Oaks, PA, USA) with a calibrated detection range of 0–5 ppm, accuracy of ±5% and sensitivity of 1% for the sensor (00-1005) and model used (C16-1). The exhaust air system servicing the unit and the treated room was kept operational at all times. Inside the sterilization room the exhaust dampers were sealed but could be opened to allow rapid evacuation of the ClO_2_ gas in the event of an emergency.

Initially chlorine dioxide gas (0.1 ppm) was measured in the adjacent hallway directly in front of the treated room. The seal around the entry door to the room was adjusted and a safe zone was established 3 meters from the door. Gas monitoring of the hospital floor below the NBPCU treated room was conducted and no ClO_2_ gas was detected. 

### 2.5. Sterilization Parameters

Six separate sterilization cycles were performed and ClO_2_ concentrations of 351 ppm to 385 ppm (Table 2) were maintained in the hospital room for <3 h with no impact on areas outside the unit. Relative humidity was raised and maintained to 50% and 65% and maintained for 30 min prior to each sterilization cycle using a portable steamer controlled from outside the room. Chlorine dioxide exposures of 677 ppm-h to 890 ppm-h were achieved in the hospital room. Four sterilization cycles were performed with the interior hospital room doors, the bathroom door, and the cabinet door open. Two cycles evaluated the impact of closed bathroom and cabinet doors on sterilization. 

### 2.6. Organism Processing

Following completion of each sterilization cycle and reduction of ClO_2_ gas in the room to 0 parts per million, test and control organisms were retrieved from placement sites and transported in a hard sided container to a biosafety level 2 laboratory for processing. Organisms were collected from quad plates using standard swabbing technique previously reported and swabs were transferred to 700 μL phosphate buffer saline (PBS) [22,23]. PBS swab solutions were serial diluted, plated onto culture media, incubated, and evaluated for colony forming unit plate counts. Log reduction in viable organisms exposed to ClO_2_ was assessed through comparison of serial dilution plate counts of the non-exposed control organisms. Control samples were processed using the same protocols as exposure samples. Geometric mean log reductions and percent inactivation were calculated for each organism. Statistical significance was not calculated as complete inactivation for all or most samples of each organism resulted in limited variance. Complete inactivation allows scrutiny of growth or no growth of specimens at placement sites to distinguish differences between treatment protocols. For these reasons log reduction results are reported as mean log reduction of all 10 sites throughout the room to indicate overall penetration of ClO_2_ throughout the hospital room.

**Table 2:** Organism inactivation following exposure to chlorine dioxide gas in a hospital environment.

ClO_2_ Conc. (ppm)	Exposure (ppm-h)	% RH	Mean Log Reduction (% inactivation) of all 10 placement sites ^†^							
			*A. baumannii* HDR BC 9782	*E. coli* ATCC 25922	*E. coli* ATCC 51446	*E. faecalis* ATCC *29212*	*E. faecalis* ATCC 51295	*M. smegmatis* ATCC 14468	*S. aureus* ATCC 25323	*S. aureus* ATCC 43300
351	677	50	9.03 (100%)	9.96 (100%)	10.00 (100%)	10.1 (100%)	9.96 (100%)	10.0 (100%)	8.48 (100%)	9.04 (100%)
377	890	65	7.65 (100%)	8.66 (100%)	9.13 (100%)	8.28 (100%)	9.06 (100%)	8.91 (100%)	7.41 (100%)	8.27 (100%)
379	767	65	9.33 (100%)	9.02 (100%)	9.35 (100%)	9.40 (100%)	9.21 (100%)	9.22 (100%)	8.41 (100%)	8.69 (100%)
385	770	65	8.24 (100%)	8.93 (100%)	9.03 (100%)	8.03 (100%)	9.04 (100%)	9.08 (100%)	8.37 (100%)	8.54 (100%)
376	788	64	8.04 (98%)	8.93 (98%)	9.01 (98%)	8.24 (99%)	9.24 (97%)	8.97 (99%)	8.42 (99%)	8.06 (98%)
378	781	66	9.02 (98%)	9.75 (97%)	9.71 (97%)	10.07 (100%)	9.82 (98%)	9.76 (97%)	9.90 (99%)	9.93 (99%)

**^†^** Site No. 6 was the only placement site with growth following ClO_2_ exposure.

## 3. Results and Discussion

Sterilization cycles 1, 2, 3 and 4 achieved complete inactivation of duplicate pairs of *A. baumannii*, *E. coli*, *E. faecalis*, *M. smegmatis* and *S. aureus* as well as *B. atrophaeus* spores at all 10 placement sites throughout the hospital room (Table 2). This resulted in a 7.4 log to 10.1 log reduction in viable counts.

Sterilization cycles 5 and 6 (Table 2), evaluating the impact of closed doors within the hospital room completely inactivated all test organisms and spore strip indicators (Table 3) at placement sites within the primary area of the hospital room, but did not result in complete inactivation of organisms or indicators at placement site 6, which was located on the sink in the hospital room bathroom. Despite the lack of complete inactivation, a >6 log reduction of each organism was achieved in spite of the closed doors (Table 2). Of note, complete inactivation was observed for all organisms and spore strip indicators (Table 3) at placement site 7 located inside a closed metal cabinet.

**Table 3:** *Bacillus atrophaeus* biological indicator inactivation following exposure to chlorine dioxide gas.

ClO_2_ Conc. (ppm)	Exposure (ppm-h)	% RH	*B. atrophaeus* spores	
			No. of sites with growth	No. of sites without growth
351	677	50	0	10
377	890	65	0	10
379	767	65	0	10
385	770	65	0	10
376	788	64	1	9
378	781	66	1	9

The importance of the hospital environment in the transmission of pathogens associated with healthcare infections has been demonstrated and emphasizes the need for adequate methods for room disinfection [2,3,4,5,6,7,8,9]. Other modalities of decontamination, including ozone and hydrogen peroxide vapor to reduce nosocomial organisms in the hospital environment have also been evaluated [10,11,12,13,14,15,24,25,26,27,28,29]. Moat *et al.* found ozone capable of producing up to 3 log reduction of *Clostridium difficile*, *Escherichia coli*, *Staphylococcus aureus*, and *Enterococcus faecalis* in a laboratory chamber and a mock hospital room [24]. Berrington and Pedler observed that ozone decontamination in a hospital room reduced levels of MRSA contamination in close proximity to the ozone generator, but failed to produce reduction in viable bacteria at contamination sites located away from the generator [25]. French *et al.* compared the reduction of MRSA environmental contamination following manual environmental cleaning with solution of detergent sanitizer GWP 4L (GWP Group, Elland, W. Yorkshire, UK) and hydrogen peroxide vapor fumigation throughout a hospital in a study co-published with Bioquell (Hampshire, UK) [11]. Environmental levels of MRSA contamination prior to manual cleaning with detergent solution were identified at 70% of tested sites and remained at 66% of test sites following manual cleaning and 1.2% of sites following hydrogen peroxide fumigation. Of note, French observed 100% inactivation of 10^6^ *Bacillus stearothermophilus* spores from a biological indicator for gaseous decontamination. Persistence of environmental MRSA despite no growth results from 10^6^ biological indicators suggest commercially available biological indicators may not provide adequate validation compared to environmental sampling to confirm complete reduction of environmental contamination. A variety of parameters should be considered when choosing a decontamination technique, including organism concentration, contamination of varied surfaces, the presence of wet contaminated surfaces, organic contamination, and the presence of organic soil.

## 4. Conclusions

This study provides evidence that ClO_2_ gas can be used to effectively eliminate environmental bacterial contamination in the healthcare setting. Limitations of this pilot study include that engineering controls of the NBPCU hospital room were more robust than a standard hospital room. By design, the clinical setting chosen for this assessment maximized safety measures. These additional safety measures may not apply in a non-BPCU setting with rooms that are not separately sealed and do not have the capability to rapidly evacuate gas if needed. ClO_2_ has a permissible exposure limit of 0.1 ppm and a short term exposure limit of 0.3 ppm set by the U.S. Occupational Safety and Health Administration (OSHA). Chlorine dioxide gas produces mucosal eye and respiratory irritation at concentration levels of 5 ppm. Any usage of ClO_2_ should also be undertaken after a careful safety analysis, as was done in this study. In spite of these limitations, this study demonstrates that ClO_2_ may provide a viable option for decontamination of bacterial organisms. Further studies are necessary to validate the utility of gaseous ClO_2_ as an infection control modality in the healthcare setting.
